# Revised Genome Sequence of the Purple Photosynthetic Bacterium *Blastochloris viridis*

**DOI:** 10.1128/genomeA.01520-15

**Published:** 2016-01-21

**Authors:** Lu-Ning Liu, Matthew Faulkner, Xuan Liu, Fang Huang, Alistair C. Darby, Neil Hall

**Affiliations:** Institute of Integrative Biology, University of Liverpool, Liverpool, United Kingdom

## Abstract

*Blastochloris viridis* is a unique anaerobic, phototrophic purple bacterium that produces bacteriochlorophyll *b*. Here we report an improved genome sequence of *Blastochloris viridis* DSM133, which is instrumental to the studies of photosynthesis, metabolic versatility, and genetic engineering of this microorganism.

## GENOME ANNOUNCEMENT

*Blastochloris* (formerly *Rhodopseudomonas*) *viridis* is a Gram-negative purple nonsulfur photosynthetic bacterium that preferentially grows photohetrotrophically. The photosynthetic machinery of *B. viridis* has been extensively studied, comprising solely a single light-harvesting complex surrounding a photosynthetic reaction center (forming an RC-LH1 core complex) (1), as well as cytochrome *bc*_1_ and ATPase. Unlike many of the other photosynthetic bacteria, *B. viridis* uniquely produces bacteriochlorophyll *b* (2, 3). The reaction center has been determined using X-ray crystallography (4). The organization of RC-LH1 core complexes in the photosynthetic membrane has been characterized using atomic force microscopy (5, 6). Recently, the genome sequence of *B. viridis* has been reported (Data Bank of Japan accession number AP014854) (7). However, sequence analysis shows that the published genome sequence contains two large sequence segments (over 136 kbp with 130 open reading frames) that are originally from a cyanobacterium, *Geminocystis* sp. strain NIES-3709 (8), likely due to contaminations during genomic DNA preparation. Here, we report the revised complete genome sequence of *B. viridis* DSM133 and improved annotation.

Genomic DNA from *B. viridis* DSM133 was extracted using phenol-chloroform standard procedure. Whole-genome sequencing was performed with the Pacific Bioscience RS II platform using P6C4 chemistry by the Centre for Genomic Research, University of Liverpool. A total of 22,943 reads-of-insert were generated with an *N*_50_ length of 2.719, giving 89.1-fold coverage of the genome. The complete chromosome, without gaps, was assembled/polished using HGAP (9) and annotated using PROKKA 1.7.2 (10). Comparative analysis between sequenced genomes was performed and visualized using the Artemis comparison tool (11).

The genome of *B. viridis* DSM133 consists of a single circular chromosome containing 3,723,225 bp with 3,260 predicted open reading frames, 53 tRNA genes, and 9 rRNA operons. The average length of each coding sequence is 971 bp, with a total coding percentage of 85%. The genome has a G+C content of 67.9%. The photosynthetic apparatus is encoded by a number of genes organized in a photosynthesis gene cluster. Six of these genes, *pufBALMC* and *puhA,* encode the RC-LH1 core complex (light-harvesting protein β and α subunits and reaction center L, M, and H subunits, respectively). Bacteriochlorophyll biosynthesis is encoded by a gene cluster (*bchPGFNBHLMIDCXYZ*). Another gene cluster, *petABC*, encodes the cytochrome *bc*_1_. A single gene (*cycA*) encodes cytochrome *c*_2_ that is a soluble electron carrier between cytochrome *bc*_1_ and the photosynthetic reaction center. The F_0_F_1_-ATPase is encoded by two gene operons, *atpHAGDC* for the F_1_ sector and *atpIBEXF* for the F_0_ sector. In addition, there are carbon fixation-related genes for ribulose bisphosphate carboxylase (RuBisCO) (*cbbL*, *cbxSC*), RuBisCO expression protein (*cbbX*), RuBisCO-like protein 2 (*rlp2*), and carbonic anhydrase (*cynT*).

The number of endogenous plasmids in purple bacteria varies between species: no plasmid found in *B. viridis*; one plasmid identified in *Rhodopseudomonas palustris* CGA009 (12) and *Rhodobacter capsulatus* SB1003 (13); two plasmids in *Rhodobacter sphaeroides* WS8N (14), KD131 (15), and *Rhodovulum sulfidophilum* W4 (16); five plasmids in *R. sphaeroides* 2.4.1 (17) and ATCC 17025 (18).

### Nucleotide sequence accession number. 

The revised complete genome sequence of *Blastochloris viridis* DSM133 has been deposited at DDBJ/ENA/GenBank under the accession number LN907867.
